# An Exploration of Industry Expert Perception of Equine Welfare Using Vignettes

**DOI:** 10.3390/ani7120102

**Published:** 2017-12-20

**Authors:** Cordelie DuBois, Helen Hambly-Odame, Derek B. Haley, Katrina Merkies

**Affiliations:** 1 Department of Animal Biosciences, University of Guelph, Guelph, ON N1G 2W1, Canada; cdubois@uoguelph.ca; 2 Campbell Centre for the Study of Animal Welfare, University of Guelph, Guelph, ON N1G 2W1, Canada; dhaley@uoguelph.ca; 3 School of Environmental Design and Rural Development, University of Guelph, Guelph, ON N1G 2W1, Canada; hhambly@uoguelph.ca; 4 Department of Population Medicine, Ontario Veterinary College, University of Guelph, Guelph, ON N1G 2W1, Canada

**Keywords:** equine industry, vignette, equine welfare, domestic horse, online survey

## Abstract

**Simple Summary:**

Short scenarios were used to examine how equine professionals evaluated potentially welfare-compromising situations as part of a larger survey project. Participants were asked to score scenarios based on perceived severity, justify their answer, and provide an explanation for what might cause a person to put the horse in that situation. Based on the answers provided, it was determined that the duration of the situation and extent of its consequences most greatly impacted scoring values, though a large variation in score values was seen. Results from this study suggested that professionals were most sensitive to situations that had the potential to cause horses pain, which is likely to influence how they perceive and react to horses experiencing a state of poor welfare. Overall, responses from the vignettes allowed for a picture of welfare perception based on personal values.

**Abstract:**

As part of a larger Delphi survey project, equine professionals (*n* = 14) were presented with twelve short scenarios in which a horse’s welfare could be compromised. They were asked to rank each scenario (with 0 indicating no welfare concerns and 5 indicating a situation where immediate intervention was necessary), provide justification for their ranking, and give examples of what might have been the motivation behind the scenario. The wide range within vignette scores demonstrated the diversity of opinion even among a relatively small group of equine professionals. Qualitative analysis of responses to vignettes suggested that respondents typically ranked situations higher if they had a longer duration and the potential for greater or longer-lasting consequences (e.g., serious injury). Respondents were also the most sensitive to situations in which the horse’s physical well-being (e.g., painful experience) was, or could be, compromised. Financial reasons, ignorance, and human convenience were also areas discussed as potential motivators by survey respondents. Overall, responses from the vignettes allowed for a picture of welfare perception based on personal values.

## 1. Introduction

The revision of the National Farm Animal Care Council’s Code of Practice for the Care and Handling of Equines [1] in 2013 was an important milestone for the Canadian equine industry. This introduction of national standards, however, brought to light the paucity of data regarding horse welfare within the industry itself. Animal welfare, or the quality of an animal’s life as determined by its physical and mental state [2], is a multifaceted issue, and one that has received considerable attention and debate. With respect to horses, reviews of equine specific animal-based indicators (such as Dalla Costa [3]) highlight the multitude of ways that equine welfare can be compromised, including both physiological and psychological harm. Knowing the extent to which an issue affects an animal, however, is only one part of addressing welfare issues. Without knowledge of the most prevalent welfare risks within an industry, and the human motivations behind them, both improving welfare and directing scientific inquiry is a challenging task. Additionally, understanding perceptions is necessary to help facilitate behavioural change, especially when change comes at an increased cost to animal owners [4]. To combat this, exploratory qualitative work can be used to better understand the industry through the insights of those involved in it.

The use of qualitative techniques has the benefit of collecting and examining diverse opinions, as is the case of a topic such as animal welfare. To allow for productive discussions, researchers have used survey techniques such as the Delphi method (created originally by the Rand Corporation; [5]) to better facilitate the exchange and refinement of ideas [6]. Using multiple rounds, this technique gathers information and then re-presents it to a collection of experts in order to reach a consensus [6]. It has been used successfully to discuss animal welfare in multiple species, including equids (e.g., [7,8,9,10]. In combination with the Delphi’s open-ended or theoretical questions, the use of vignettes–short, two to three sentence long scenarios—allows respondents to discuss their opinions regarding what would occur “in practice” [11]. In this manner, vignettes have been used extensively in the medical literature (see review by Bachmann et al. [12]), with special focus on how clinicians evaluate potentially ill patients and what symptoms they rely on to make medical judgements. The complexity of decision-making can be explored more thoroughly using vignettes than relying on answers given in survey responses. This combination approach has been used by Collins et al. [8] to examine welfare perception in the Irish equine industry, where participants were asked to score scenarios for perceived acceptability and indicate potential motivators. Authors noted that the vignettes in particular were useful in stimulating discussion of potentially sensitive subjects [8].

This project was part of a larger Delphi survey examining the perception of welfare within the Canadian equine industry by professionals, utilizing similar methods as outlined in [8]. The aim of this portion was to use vignettes to provide additional insight into how animals are evaluated and what situations were considered the most “welfare-compromising.” In addition, we sought to determine what equine professionals believed were the causes of these situations in order to help identify areas within the industry in which change is needed. Equine professionals represent a population of industry participants who not only have a role as stakeholders but also as potential educators of other participants. Understanding their attitudes and insights with respect to equine welfare in the Canadian industry is, therefore, extremely valuable to direct future research and educational outreach.

## 2. Materials and Methods

This study was approved by the University of Guelph Research Ethics Board in compliance with federal guidelines for research involving human participants (REB #15DC024). Survey respondents (*n* = 14) were individuals who were participating in a Delphi survey examining equine professional perception of welfare issues within the Canadian industry. Vignettes were presented in the third round of this Delphi study to all participants who had completed the two previous rounds. Respondents were originally invited via email or telephone from a variety of professional groups across Canada (215 individuals contacted) to participate in a survey hosted on an online survey platform provided by Qualtrics (© 2016 Qualtrics LLC, Provo, UT, USA). The respondent pool contained at least one individual from the following professional groups: veterinarians, farriers, racing industry, certified equine massage therapist, equine dentists, certified equine riding coach, equine research, individuals with an equine-related post-secondary degree, and humane officers with experience in equine cruelty. Potential respondents were chosen based on their certification in their profession, their experience in the industry (more than ten years), and the availability of contact information (publically available).

In previous rounds of the Delphi study, the survey respondents provided and ranked a list of welfare issues they perceived to be a threat to the Canadian equine industry. These issues were then used as the basis for the creation of short scenarios (“vignettes”). Survey respondents were presented with 12 vignettes in which a horse’s welfare may or may not have been compromised (full list of vignettes in Appendix A). Respondents were asked to rank each situation on a scale of 0 (welfare is not compromised) to 5 (distress that requires immediate intervention), explain their chosen answer, and provide possible motivators for the situation. Data were exported from Qualtrics into Microsoft Excel for analysis. Median scores and interquartile ranges (IQR) were calculated for each vignette. All open-ended questions were coded by C. DuBois using QSR International’s NVivo 10 qualitative data analysis Software (QSR International Pty Ltd. Version 10, 2012, Burlington, MA, USA) Data was coded using single summary words (“descriptive coding”; e.g., “financial”) or a phrase from the written material (“in-vivo coding”; e.g., “desire to win”) as sorting categories. All coding categories were drawn directly from the data itself. The number of coding references were then tabulated.

## 3. Results

**Figure 1:**
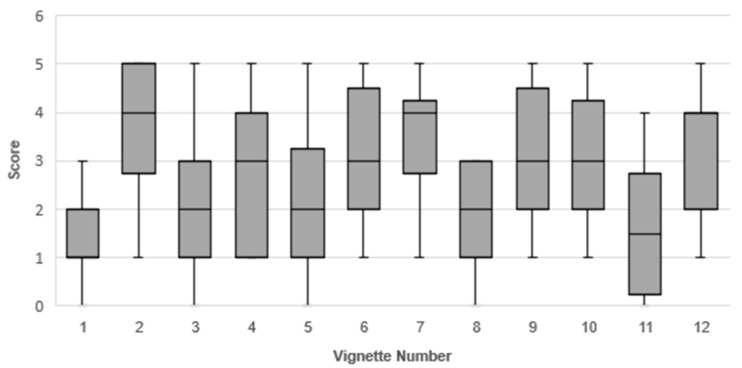
Box and whisker plot of vignette (twelve numbered scenarios) scores as assigned by survey respondents (*n* = 14). Vignettes were scored between 0 (welfare is not compromised) and 5 (distress that requires immediate intervention).

Vignettes were assigned a score between 0 and 5 by each respondent (median scores presented in Figure 1). Vignettes 2 (oral analgesic to “mask” lameness), 7 (frozen water trough in winter), and 12 (human antacids for potential stomach ulcers) received the highest median scores (4), while Vignettes 1 (isolating a horse for sale), 3 (“tuning up” a pony), and 8 (accidental breeding) received the lowest median scores (1, 2, and 2, respectively).

**Figure 2:**
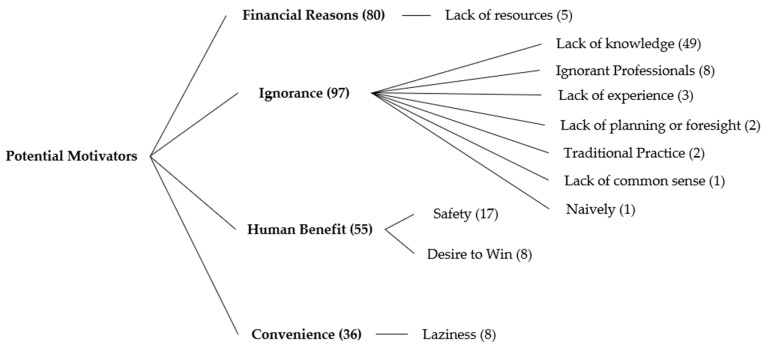
Taxonomic tree diagram of the four highest referenced potential motivators behind the welfare concerns identified in 12 vignettes by respondents (*n* = 14). Numbers in brackets indicate number of references for each category.

**Figure 3:**
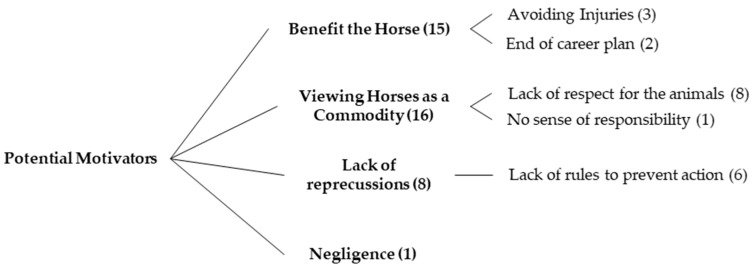
Taxonomic tree diagram of the four lowest referenced potential motivators behind the welfare concerns identified in the vignettes by respondents (*n* = 14). Numbers in brackets indicate number of references for each category.

Survey respondents provided the reasoning behind their chosen score, with 75 references indicating that a situation warranted more concern if there were serious consequences (either immediately or in the long-term), paying particular attention to pain (58 references all vignettes represented). Participants indicated that they offered low scores or moderate scores if they felt that different horses would react to the situation differently (12 references), if they needed more medical information (e.g., body condition score) about the horse to score properly (19 references), or if the outcome of the situation (e.g., owner trimming their own horse’s hooves) was dependent on the owner’s skill level (19 references). Situations were also framed as either short-term welfare concerns (5 references) or long-term welfare concerns (21 references). Of the 45 references where survey respondents indicated that they did not feel welfare was significantly compromised, Vignette 2 was the only scenario in which no participant indicated there was not a welfare concern. In addition to the comments describing potential welfare issues, there were seven references to human safety in relation to the scenarios, 11 references to ethical issues, and 13 references in which the respondent indicated the person in the scenario was acting in place of a professional. Survey respondents also identified eight thematic categories of potential motivators behind the scenarios, indicated in Figure 2 and Figure 3. 

## 4. Discussion

Using short vignettes, this study explored the ways in which equine professionals evaluated equine welfare when presented with a “snapshot” of a horse in a potentially welfare-compromising situation. The breadth of professional opinion on what constitutes a situation of welfare concern was demonstrated by the large spread of the IQRs for each scenario (Figure 1). There were several instances where one respondent would indicate welfare was severely compromised (a score of 5), while another would state that welfare was not compromised (a score of 0). Indeed, only one vignette (#2—masking lameness) did not receive any comments regarding the perceived acceptability of the practices described. Respondents also expanded on the scenarios, indicating ways in which welfare could be more or less compromised depending on the fine details of the scenario, which may have resulted in the variability of final scores. 

Variability in responses and scores may also have been due to the respondents’ familiarity with the situation, as seen in Horseman et al. [13] where survey respondents were more critical of potential welfare-compromising situations they were less familiar with. There were three instances where respondents indicated that the situation described was considered “common practice”, and thus did not warrant intervention. Additionally, the small but diverse sample size likely further increased variability in answers, resulting in vastly different opinions on what constituted a situation requiring immediate intervention. Respondents also justified their score based on the potential the situation had to cause repercussions in the long term, or noted that the situation was poor in the short term but would resolve itself quickly and therefore deserved a lower score (e.g., horse isolated for sale purposes). In that respect, it appeared that some respondents were willing to compromise welfare in the short term for the sake of human needs. The consequences were also (with one exception) carefully considered, with special attention paid to situations that would cause the horse long-lasting pain or injury. The qualitative responses of respondents suggested that they had difficulty making accurate judgements regarding the scenarios with the limited information given, particularly scenarios involving a medical aspect (e.g., an injury). In the future, shorter vignettes may be more useful in determining what aspect of welfare is potentially being compromised (as seen in [8]); however, more detailed scenarios may help determine the point at which a situation no longer becomes acceptable.

Scenarios involving drug use had the highest median scores. In Vignette 2, use of an analgesic to mask lameness received a median score of 4, though whether this was related to the drug use or the combination of drug use and a horse perceived to be in pain was unclear. It seems likely that the additional element of pain increased the score, as in Vignette 9 where a horse was given a sedative to improve its performance, the median score was only 3. Comparatively, the scenario in which a horse is thought to have stomach ulcers and is receiving human antacids also achieved a median score of 4. More scenarios involving different types of drugs may have been able to more accurately reflect the perception of drug use or abuse specifically, without the confound of recognizable pain. Though “unwanted horses” is an issue of importance [14], the scenario which resulted in an unplanned horse birth received the lowest scores. Survey respondents indicated that the unwanted horse scenario was not in and of itself “welfare-compromising” until the horse in question received the full effects of being “unwanted” (e.g., being sent to slaughter and potentially suffering the associated welfare risks). While respondents indicated in other vignettes that they were conscious of long-lasting effects of certain aspects of the scenarios, it was clear that the concept of overpopulation was difficult to capture in a short vignette. If a similar approach to this study is used in the future, scenarios involving different aspects of overpopulation (e.g., the horse breeder, the person who buys horses to resell them) may illicit different responses and give a better understanding of at what point overpopulation becomes a welfare concern.

With a total of 58 open comment references, survey respondents appeared to most easily determine if a horse was (a) currently suffering or (b) at-risk of suffering as a result of the scenario (e.g., potential injury). In contrast, only one reference was made to the possibility of the horse’s ability to perform natural behaviours being compromised. It is possible that the vignettes over-represented scenarios where a horse’s physical health may have been affected, or that survey respondents were more attuned to threats to the horse’s physical health. Though half of the vignettes did contain an element of potential psychological distress, the scenario in which the horse was isolated had the lowest median score. From the comments, it suggested that some survey respondents believed this scenario to be a short term one which may have reduced the score. Vignettes similar to those used by Collins et al. [8] which first asked respondents to identify what aspect of welfare was being compromised may have helped to determine if respondents were more sensitive to situations involving an animal in physical pain.

“Ignorance” and “financial reasons” were the two most frequently identified motivators, both being listed as a potential motivator for every vignette. They have also been previously identified as potential motivators behind welfare-compromising situations in the Irish [8] and English [15] equine industries. Participants indicated that it was a lack of knowledge, primarily, that directed behaviour, a finding supported by Horseman et al. [13] who noted that owners were not consulting the appropriate professionals or else not seeking advice at all when in need of information. Many participants indicated that the outcome of five scenarios was dependent on the skill of the person performing the action. Finally, no respondent cited willful abuse as a potential motivator behind the actions in any scenario. This further supports the idea that respondents believe that compromised equine welfare is more commonly the result of reasons (predominantly ignorance) other than the intention to willfully cause harm to the animal. 

Survey respondents also indicated instances where the choices of the people in the scenarios could have benefited the horse in certain aspects or resulted in increased human safety with a relatively low “penalty” for the horse. These types of motivators did appear to have an effect on the scenario’s score, as survey respondents frequently justified a slight decrease in the welfare of the horse in exchange for a greater increase in areas such as human safety (e.g., in the scenario in which a horse is given a tranquilizing drug before competition). Opinions were divided in some cases: for example, for some scenarios, some survey respondents indicated human safety was being improved, while other respondents indicated that the safety of both human and horse was being compromised. The compromising of welfare, especially in the short term, was also cited as a reason for the low score in Vignette 1, where some participants indicated that the social isolation could improve the horse’s chances of sale, and thus shorten the duration of the scenario. In future surveys, it may be possible to better determine what welfare tradeoffs are acceptable by using more specific scenarios or cut-off points. 

Of additional note, survey respondents commented on both the welfare implications and the ethical implications of several scenarios, which is a topic worth further investigation. Vignettes 2, 3, 9, and 11 were identified by at least one participant as being ethically concerning, in some cases regardless of any potential welfare concerns the participant had. Though this project did not focus intentionally on the ethics of any of the vignettes, the role ethics plays in determining welfare values may be worth exploring in future surveys. 

## 5. Conclusions

The diversity of opinions and values further supports the complexity of perceptions of welfare at the situational level. Results from this survey suggest that Canadian equine professionals judge potentially welfare-compromising situations based on their duration and the magnitude of their consequences. Despite differences in scores, however, they consistently report ignorance and financial reasons as the motivators behind all vignettes. Offering equine professionals the opportunity to comment on “practical” equine welfare scenarios helps further the understanding of what issues are perceived as being “better” or “worse” for the animals involved, allowing for more nuanced answers than asking broader questions regarding equine welfare issues. 

## Appendix A

**Table A1:** Vignettes viewed by survey respondents (*n* = 14).

Vignette #	Scenario
1	A horse owner is looking to sell their horse. In order to ensure that he doesn’t get “marked up” by his pasture mates, his owner turns him out alone in a paddock where he can still see other horses but cannot interact with them, even over the fence.
2	A horse owner takes their horse to a local horse show run by their uncle, where both the owner and their younger sibling will be competing. Their horse finishes the first class and seems to be limping. The owner gives their horse an oral analgesic so that their sibling can ride the horse in a class later the same day.
3	A person owns a riding stable with several ponies. One spirited pony has been off work for several weeks and the owner wants to use him in a beginner’s lesson. They ask one of their more experienced riders to “tune him up” before the lesson.
4	A horse owner notices their horse’s feet need trimming and decides to do it themselves, as they have watched their farrier do it many times before.
5	A person’s horse is known for “mixing” her hay into her bedding every night. In order to stop this behaviour, her owner reduces the amount of hay she is given while she’s in her stall.
6	A couple’s two children leave home for university. As they live on a farm, the couple decides to leave their children’s two horses on the back pasture instead of stabling them inside at night. They check on the horses every two to three days to ensure they have hay and water.
7	A person’s horses stay outside all winter and often their water trough freezes over when the temperature drops. Their owner carries two buckets of warm water out to help thaw the troughs in the morning and evening, but otherwise does not provide additional water.
8	A person boards horses at their hobby farm. A client brings a mare with a colt, and after assurances that he’s too young to breed, the hobby farm owner turns them out together with one of their mares. By the next spring, their mare gives birth to an unexpected foal.
9	A riding school owner often takes their horses off-property to horse shows where they are ridden by students. On show days, the riding school owner gives their horses an injection because it “settles them down” for the inexperienced riders.
10	A person has a young horse that they would like to be able to ride. They decide to start the horse under saddle by themselves after watching a YouTube series produced by a popular horse trainer.
11	A person’s horse develops a career-ending injury during her final competition performance. The horse’s owner decides to retire her as a brood mare because they think she would make a wonderful mother. The mare is bred upon her return to the farm.
12	A person thinks their horse has stomach ulcers. At the recommendation of their trainer, they give their horse 15 over-the-counter antacid pills (for humans) per day until her symptoms disappear.
